# Supplementary material for: Deletion of wfs1 Impairs Oligodendrocyte Precursor Cells Dorsal Distribution and Myelination Through the wfs1-hmgcs1 Axis in Zebrafish
Source: Biology (Basel). 2026 Aug 16;15(16):1402. doi: 10.3390/biology15161402 (PMC13510151; doi:10.3390/biology15161402)
Supplement: Supplementary file 1 [file biology-15-01402-s001.zip › File S1-Transcriptome sequencing data and differentially expressed genes (DEGs) in wfs1-- zebrafish at 5 dpf/1.GO/wfs1KOvsWT/GSEA/WT/UBIQUITIN_BINDING/UBIQUITIN_BINDING(GO_0043130).html]

Details for gene set UBIQUITIN\_BINDING(GO:0043130)[GSEA]


|  || Dataset | deg.sample |
| Phenotype | sample.cls |
| Upregulated in class | WT |
| GeneSet | UBIQUITIN\_BINDING(GO:0043130) |
| Enrichment Score (ES) | -0.44615144 |
| Normalized Enrichment Score (NES) | -1.7763078 |
| Nominal p-value | 0.0 |
| FDR q-value | 0.2990162 |
| FWER p-Value | 0.717 |
Table: GSEA Results Summary

  

Fig 1: Enrichment plot: UBIQUITIN\_BINDING(GO:0043130)
  
Profile of the Running ES Score & Positions of GeneSet Members on the Rank Ordered List

  

| PROBE | DESCRIPTION   (from dataset) | GENE SYMBOL | GENE\_TITLE | RANK IN GENE LIST | RANK METRIC SCORE | RUNNING ES | CORE ENRICHMENT || 1 | 393973 | rae1 |  |  | 554 | 2.834 | 0.1121 | No |
| 2 | 768297 | cuedc1a |  |  | 5922 | 0.595 | -0.0292 | No |
| 3 | 563175 | smarcad1b |  |  | 7962 | 0.426 | -0.0738 | No |
| 4 | 569134 | ubxn11 |  |  | 8810 | 0.380 | -0.0830 | No |
| 5 | 415200 | zgc:86839 |  |  | 10430 | 0.277 | -0.1212 | No |
| 6 | 558048 | n4bp2 |  |  | 11335 | 0.225 | -0.1393 | No |
| 7 | 100537478 | ubxn2a |  |  | 11421 | 0.221 | -0.1319 | No |
| 8 | 334682 | wdr48b |  |  | 11831 | 0.199 | -0.1356 | No |
| 9 | 406442 | amfr |  |  | 12054 | 0.188 | -0.1340 | No |
| 10 | 445284 | rad23aa |  |  | 12484 | 0.165 | -0.1399 | No |
| 11 | 327327 | n4bp1 |  |  | 13193 | 0.131 | -0.1562 | No |
| 12 | 494163 | tab2 |  |  | 13529 | 0.114 | -0.1615 | No |
| 13 | 336876 | tollip |  |  | 13962 | 0.094 | -0.1707 | No |
| 14 | 403012 | bub3 |  |  | 14162 | 0.084 | -0.1731 | No |
| 15 | 492805 | nsfl1c |  |  | 14202 | 0.082 | -0.1706 | No |
| 16 | 445020 | pdcd7 |  |  | 14286 | 0.079 | -0.1696 | No |
| 17 | 556847 | cuedc1b |  |  | 14294 | 0.078 | -0.1663 | No |
| 18 | 327447 | nploc4 |  |  | 14623 | 0.064 | -0.1736 | No |
| 19 | 569903 | shrprbck1r |  |  | 14645 | 0.063 | -0.1714 | No |
| 20 | 324654 | aup1 |  |  | 14732 | 0.059 | -0.1714 | No |
| 21 | 393183 | ubap1 |  |  | 15751 | 0.009 | -0.2030 | No |
| 22 | 337168 | nbr1a |  |  | 17247 | -0.043 | -0.2480 | No |
| 23 | 562839 | rad23ab |  |  | 17912 | -0.072 | -0.2656 | No |
| 24 | 566547 | tab3 |  |  | 17994 | -0.075 | -0.2647 | No |
| 25 | 541415 | zgc:101577 |  |  | 17999 | -0.075 | -0.2613 | No |
| 26 | 569700 | usp16 |  |  | 18476 | -0.097 | -0.2718 | No |
| 27 | 335810 | vps36 |  |  | 19065 | -0.125 | -0.2846 | No |
| 28 | 393536 | rad23b |  |  | 20084 | -0.170 | -0.3088 | No |
| 29 | 567642 | si:dkey-181m9.8 |  |  | 20372 | -0.182 | -0.3095 | No |
| 30 | 334627 | ubr5 |  |  | 21557 | -0.244 | -0.3356 | No |
| 31 | 404050 | rnf8 |  |  | 22378 | -0.288 | -0.3481 | No |
| 32 | 393313 | ube2nb |  |  | 22491 | -0.295 | -0.3382 | No |
| 33 | 324855 | plaa |  |  | 22758 | -0.311 | -0.3323 | No |
| 34 | 334081 | rnf168 |  |  | 23936 | -0.388 | -0.3515 | No |
| 35 | 393414 | ascc2 |  |  | 23993 | -0.391 | -0.3354 | No |
| 36 | 436684 | otub1a |  |  | 24435 | -0.416 | -0.3303 | No |
| 37 | 558011 | usp13 |  |  | 26716 | -0.600 | -0.3744 | No |
| 38 | 431715 | rbck1 |  |  | 29001 | -0.920 | -0.4041 | Yes |
| 39 | 436773 | otub1b |  |  | 29318 | -1.006 | -0.3681 | Yes |
| 40 | 445199 | cks1b |  |  | 30051 | -1.266 | -0.3332 | Yes |
| 41 | 550284 | wdr92 |  |  | 30093 | -1.282 | -0.2760 | Yes |
| 42 | 100004590 | ubap1la |  |  | 30612 | -1.598 | -0.2192 | Yes |
| 43 | 796450 | adrm1 |  |  | 31153 | -2.152 | -0.1379 | Yes |
| 44 | 445403 | uspl1 |  |  | 31663 | -3.523 | 0.0071 | Yes |
Table: GSEA details
[plain text format]

  

Fig 2: UBIQUITIN\_BINDING(GO:0043130)
  
Blue-Pink O' Gram in the Space of the Analyzed GeneSet

  

Fig 3: UBIQUITIN\_BINDING(GO:0043130): Random ES distribution
  
Gene set null distribution of ES for
 **UBIQUITIN\_BINDING(GO:0043130)**
